# Spontaneous p53 activation in middle-aged C57BL/6 mice mitigates the lifespan-extending adaptive response induced by low-dose ionizing radiation

**DOI:** 10.1038/s41514-023-00123-3

**Published:** 2023-11-07

**Authors:** Masaoki Kohzaki, Keiji Suzuki, Akira Ootsuyama, Ryuji Okazaki

**Affiliations:** 1 Department of Radiobiology and Hygiene Management, Institute of Industrial Ecological Sciences, University of Occupational and Environmental Health, Kitakyushu, Japan; 2 Department of Radiation Medical Sciences, Atomic Bomb Disease Institute, Nagasaki University, Nagasaki, Japan; 3 Department of Radiation Biology and Health, School of Medicine, University of Occupational and Environmental Health, Kitakyushu, Japan

**Keywords:** Ageing, Cell signalling, Molecular biology, Proteins, Cancer

## Abstract

Understanding the biological effects of low-dose (<100 mGy) ionizing radiation (LDR) is technically challenging. We investigated age-dependent LDR effects using adaptive response experiments in young (7-to 12-week-old) and middle-aged (40-to 62-week-old) C57BL/6 mice. Compared with 3 Gy irradiation, 0.02 Gy preirradiation followed by 3 Gy irradiation prolonged life in young mice but not middle-aged mice. Preirradiation also suppressed irradiation-induced 53BP1 repair foci in the small intestines, splenic apoptosis, and p53 activity in young mice but not middle-aged mice. Young p53^+/−^ C57BL/6 mice did not show these adaptive responses, indicating that insufficient p53 function in young mice mitigated the adaptive responses. Interestingly, p53 activation in middle-aged mice spontaneously became approximately 4.5-fold greater than that in young mice, possibly masking LDR stresses. Furthermore, adaptive responses in young mice, but not in middle-aged mice, suppressed some senescence-associated secretory phenotype (SASP) factors (*IL-6, CCL2*, *CCL5*, *CXCL1*). Thus, LDR-induced adaptive responses associated with specific SASP factors may be attenuated by a combination of reduced DNA damage sensor/transducer function and chronic p53 activation in middle-aged mice.

## Introduction

It is widely accepted that the biological effects of low doses (<100 mGy) of ionizing radiation (IR) are technically difficult to determine^1^. However, understanding the biological effects of low-dose radiation (LDR), especially its long-term effects, such as aging and cancer, is of great societal significance. For example, there is a need to assess the long-term effects of increasingly sophisticated radiation therapy on medical personnel. Analyses of the adaptive responses induced by LDR followed by high-dose radiation (HDR) can produce some of the most definitive biological results^2^. This phenomenon is also called hormesis when radiation is considered one of the environmentally toxic substances, and low levels of LDR stresses followed by HDR result in a biphasic dose-response^3^. In in vivo studies using mice, the priming dose used for the adaptive response to HDR has ranged widely from 0.001 mGy to 1 Gy^4–7^. The effective time interval between the priming LDR and HDR for adaptive response ranges from 1 day to 1 week or more in young wild-type C57BL/6 mice but is only 4 h in young p53^+/−^ C57BL/6 mice^5,6^. Thus, p53 (tumor protein 53 [TP53] in humans and transformation-related protein 53 [Trp53] in mice) plays a pivotal role in the adaptive response induced by LDR. After IR exposure, p53 acts downstream of ataxia-telangiectasia mutated (ATM) in a signal transduction pathway through p53 direct phosphorylation at serine 15 (Ser15)^8^. p53 phosphorylation is essential for apoptosis induction^9^, which can aggressively eliminate cells with unrepaired DNA damage and/or mutations and cancer cells supported by the immune system. In fact, mice with mutation of Ser15 in the p53 gene spontaneously develop late-onset lymphomas and exhibit accelerated aging^10^, suggesting that p53 activity reflected by p53 phosphorylation at Ser15 is indispensable for both antiaging and tumor suppression in mammals. These p53 functions decline with age^11^ and result in accelerated cancer progression in mammals after middle age^12^. Indeed, p53^−/−^ mice die within 1 year^13,14^. p53 is activated in response to various stresses, such as DNA damage. Upstream of p53 activation, p19-ARF (p14-ARF in humans), which is encoded by the *INK4a-ARF* locus, functions in tumor suppression in a p53-dependent manner^15^. ARF can bind to MDM2 and promote MDM2 degradation^16^, and MDM2 can form a complex with p53 and inhibit p53-mediated activation^17^. Downstream of p53 activation, WAF1/p21 is a well-established mediator of p53-dependent tumor suppression^18^. These p53 regulation pathways must be tightly regulated to maintain diverse cellular processes^19^.

Some tissues, such as the spleen and intestines, are highly sensitive to IR; for example, acute radiation syndrome is caused by gastrointestinal disorders induced by the collapse of intestinal epithelial cells and hematopoietic disorders due to a lethal dose of IR^20^. In contrast, the biological effects of LDR in the spleen and intestines remain unclear. Recently, a method to detect DNA damage induced by medium-dose radiation (1 Gy) and LDR (0.1 Gy) in the intestines was established (see Methods)^21^ using a recognized DNA double strand break (DSB) marker, 53BP1^22^. This method will be useful for determining the remaining DNA damage in the intestines, which may cause chronic inflammation and carcinogenesis. The biological effects of the adaptive response are very mild, and it takes a long time to observe phenotypic output. Adaptive response experiments are thus costly and labor intensive. Therefore, there have been no systematic studies to elucidate the role of p53 expression through comparison of the adaptive responses induced by very low-dose (<100 mGy) radiation (VLDR) in young and middle-aged C57BL/6 mice.

Senescence is a tumor-suppressing process. Some senescent cells secrete many factors mainly proinflammatory cytokines (IL-1, IL-6), chemokines (CCL2, CCL3, CCL5, CXCL1, CXCL8, CXCL9, CXCL10, CXCL11, CXCL12), MMP family members (MMP1, MMP3 MMP10), inflammatory molecules (NF-κB, TNFα, TGFβ, IFNγ), growth factors (PAI-1, VEGF), and ligands^23,24^. Together, these molecules form the senescence-associated secretory phenotype (SASP), which can promote a proinflammatory microenvironment, senescence, and cancer^25^. Although the SASP can affect lifespan and although p53 has been reported to suppress the SASP^26^, adaptive responses, including changes in p53 regulatory pathways, the SASP, and immune surveillance, have not been comprehensively compared between young and middle-aged mice. Therefore, the purpose of this study was to establish a method for quantitatively analyzing adaptive responses essential for lifespan extension after radiotherapy and for radiation protection after HDR exposure in mammals and to deepen our molecular understanding of these adaptive responses. The results provide important insights for the risk assessment of workers engaged in radiation-related work, such as radiotherapy.

## Results

### Adaptive responses in young and middle-aged C57BL/6 mice at the organismal level

**Fig. 1:**
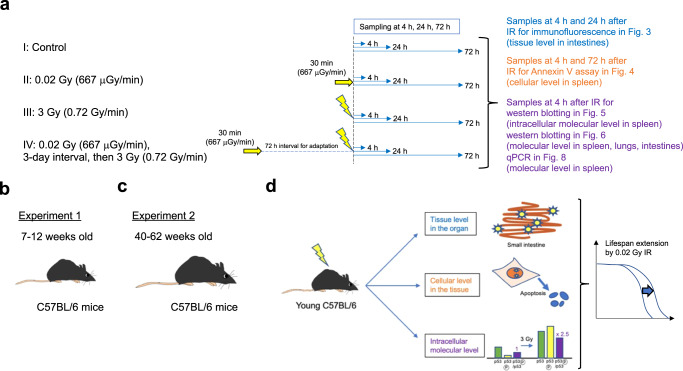
Overview of the experimental process for determining the adaptive response in C57BL/6 mice. **a** Four different conditions were set: I. Nonirradiated controls; II. 0.02 Gy (100 µGy/min)-irradiated mice; III. 3 Gy (0.72 Gy/min)-irradiated mice; and IV. 0.02 Gy-preirradiated, 72-h interval, 3 Gy-irradiated mice. The timing of sacrifice for the four groups was the same. Specifically, group IV mice were preirradiated with 0.02 Gy at 3 days before 3 Gy irradiation, and both group III mice and group IV mice were irradiated with 3 Gy on the day of sacrifice. After the last IR exposure, we collected samples at 4 h, 24 h, and 72 h and analyzed them. The experiment at the tissue level in the intestines (blue) was conducted 4 h and 24 h after the final IR exposure. The experiment at the cellular level in the spleen (orange) was conducted 4 h and 72 h after the final IR exposure. The experiment at the intracellular molecular level (purple) was conducted 4 h after the final IR exposure. The time scale is not exactly drawn. The same experiments were conducted in (**b**) 7 to 12-week-old (young) C57BL/6 mice as experiment 1 and (**c**) 40 to 62-week-old (middle-aged) C57BL/6 mice as experiment 2. **d** Comparison of these mice to assess the different biological outcomes at the tissue, cellular, and intracellular molecular levels. These outcomes can be discussed in the context of the lifespan-extending adaptive response induced by very low-dose IR.

**Fig. 2:**
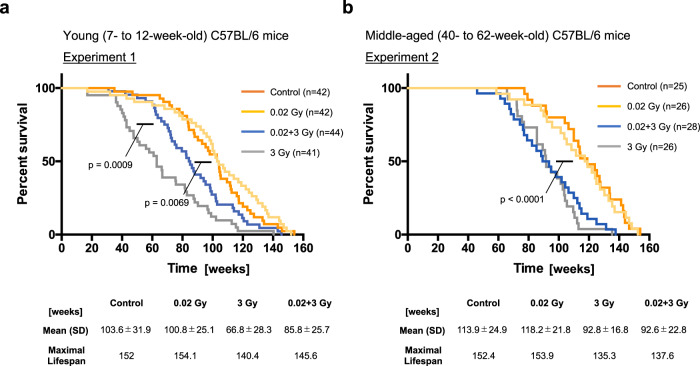
Kaplan‒Meier survival curves of experiments 1 and 2. Moribund mice were euthanized via cervical dislocation and necropsied. Gehan‒Breslow‒Wilcoxon tests were used to calculate the significance of the survival curves. Each experiment was conducted several times with different numbers of mice, and the total result is shown. Time 0 indicates the point at which the mouse was born. **a** Kaplan‒Meier survival curves of the young (7- to 12-week-old) control C57BL/6 mice (*n* = 42), 0.02 Gy-exposed mice (*n* = 42), 0.02 Gy + 3 Gy-exposed mice (*n* = 44) and 3 Gy-exposed mice (*n* = 41) are shown. The number of cohorts in each group was eight for the control group, four for the 0.02 Gy group, eight for the 3 Gy group, and four for the 0.02 + 3 Gy group. **b** Kaplan‒Meier survival curves of the middle-aged (40- to 62-week-old) control C57BL/6 mice (*n* = 25), 0.02 Gy-exposed mice (*n* = 26), 0.02 Gy + 3 Gy-exposed mice (*n* = 28) and 3 Gy-exposed mice (*n* = 26). The number of cohorts in each group was four for the control group, three for the 0.02 Gy group, five for the 3 Gy group, and four for the 0.02 + 3 Gy group.

Established adaptive response experiments have revealed that LDR exposure followed by HDR exposure yields a longer lifespan than single HDR exposure in mice. In these experiments, the timing of LDR priming prior to HDR exposure has been 24 to 168 h in wild-type C57BL/6 mice but only 2 to 4 h in p53^+/−^ mice based on the level of apoptosis and on the expression of p53 and p53-related genes in mouse splenocytes^5^. Therefore, we set 72 h as the priming time to obtain adaptive responses in group IV and as the sampling time for all groups, as previously reported (Fig. 1a). p53 function reportedly declines with age^11^. Thus, we performed two experiments, namely, experiment 1 with young C57BL/6 wild-type mice (Fig. 1b) and experiment 2 with middle-aged C57BL/6 wild-type mice (Fig. 1c), to compare the phenotypes at the tissue level within organs, at the cellular level within tissues, and at the intracellular molecular level to obtain deeper insight into the adaptive response (Fig. 1d). We reproducibly observed significant lifespan extension in young (7-to 12-week-old) C57BL/6 mice preirradiated with 0.02 Gy and then irradiated with 3 Gy compared with mice subjected only to 3 Gy irradiation (*p* = 0.0009, Fig. 2a). Importantly, prolongation of life was not observed in middle-aged (40-to 62-week-old) C57BL/6 mice (Fig. 2b). These data, obtained by the Kaplan‒Meier method, indicated that the adaptive response induced by VLDR (0.02 Gy) was diminished in middle-aged C57BL/6 mice.

### Adaptive responses in young and middle-aged C57BL/6 mice at the organ level

**Fig. 3:**
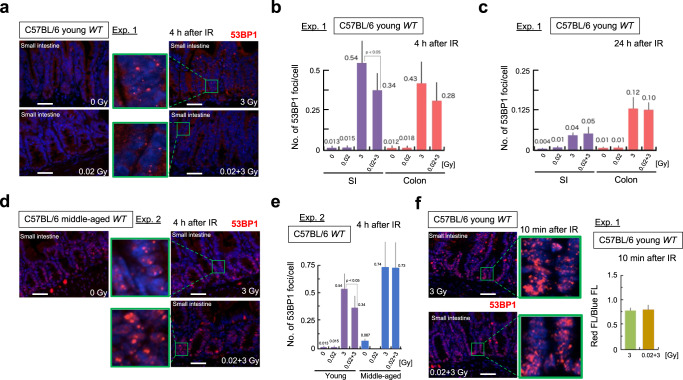
DNA damage kinetics after IR exposure in mouse intestines in experiment 1 and experiment 2. Scale bars 0.1 mm. **a** Representative images of 53BP1 foci (red) and DAPI (blue) at 4 h after IR exposure in the crypts of young C57BL/6 mice. The enlarged images are shown in green next to the original images. **b** Numbers of 53BP1 foci per cell at 4 h after IR exposure in young C57BL/6 mice. Five mice per condition were used, and data were obtained at 10 randomly selected sites. The data show the mean (s.d.). The *p* values were determined by the two-tailed *t* test. **c** Numbers of 53BP1 foci per cell at 24 h after IR in young C57BL/6 mice. Five mice per condition were used, and the data were obtained at 10 randomly selected sites. The data show the mean (s.d.). The *p* values were determined by the two-tailed *t* test. **d** Representative images of 53BP1 foci (red) and DAPI (blue) at 4 h after IR exposure in the crypts of middle-aged C57BL/6 mice. **e** Comparison of the numbers of 53BP1 foci per cell at 4 h after IR exposure in young and middle-aged C57BL/6 mice. Five mice per condition were used, and the data were obtained at 10 randomly selected sites. The data show the mean (s.d.). The *p* values were determined by the two-tailed *t* test. **f** The numbers of 53BP1 foci per cell at 10 min (immediately) after IR exposure in young C57BL/6 mice. The numbers of 53BP1 foci (red FL) per cell (blue FL) at 10 min after IR 3 Gy and 0.02 + 3 Gy in young C57BL/6 mice are shown. Five mice per condition were used, and the data were obtained at 10 randomly selected sites.

First, we examined the tissue damage induced by IR in the four groups by counting 53BP1 foci at the tissue level in the intestines (Fig. 3a)^21^. At 4 h after 3 Gy IR exposure, the small intestines (SIs) and colons of young and middle-aged mice were greatly damaged (Fig. 3b). This tissue damage promptly decreased within 24 h after IR exposure (Fig. 3c).

In young wild-type mice, the tissue damage induced by 0.02 + 3 Gy was lower than that induced by 3 Gy (Fig. 3b). Notably, such tissue damage reductions were not observed in middle-aged C57BL/6 mice (Fig. 3d, e). Moreover, the DNA damage represented by 53BP1 foci immediately (10 min) after IR exposure was induced to the same extent by 3 Gy and 0.02 + 3 Gy (Fig. 3f). Hence, the difference in the number of 53BP1 foci at 4 h after IR exposure in young C57BL/6 mice should have reflected the remaining DNA damage (Fig. 3b). Taken together, these results indicate that the DNA damage induced by 3 Gy irradiation is efficiently repaired at the tissue level in young C57BL/6 mice subjected to 0.02 Gy pre-IR by 4 h after irradiation.

### Adaptive responses in young and middle-aged C57BL/6 mice at the cellular level

**Fig. 4:**
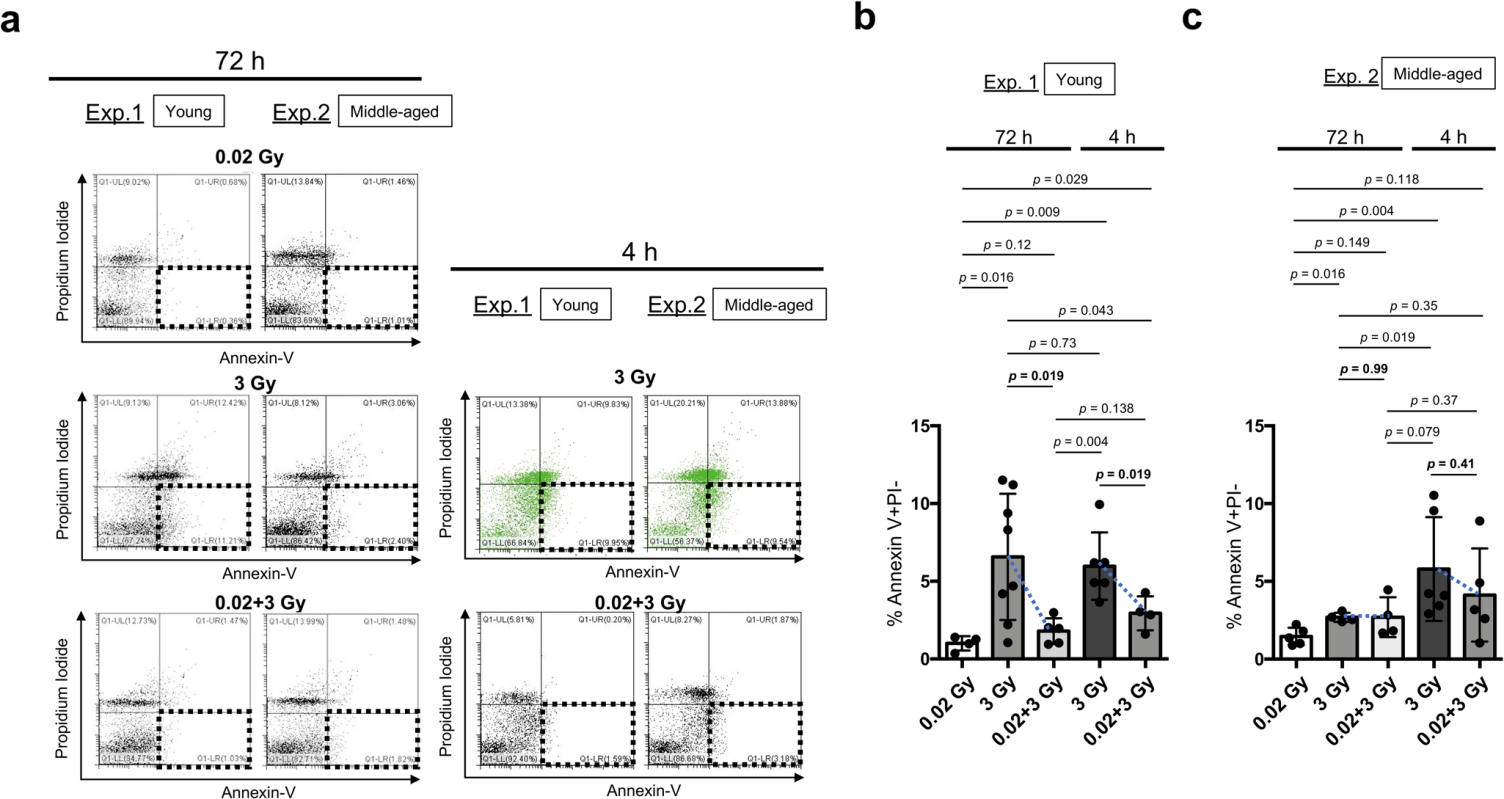
Apoptosis induction in splenocytes after IR exposure in experiment 1 and experiment 2. **a** Representative images of apoptosis induction (PI negative, Annexin-V positive; dotted squares) in all the examined groups based on FACS analysis. The apoptosis induction ratio at 72 h after 0.02 Gy irradiation was used as a control. We observed continuous apoptosis in young C57BL/6 mice 72 h after 3 Gy. To visually distinguish the adaptive response between 3 Gy and 0.02 + 3 Gy at 4 h after IR, the results for the 3 Gy-exposed group at 4 h after IR group are shown in green. **b** Percentages of apoptosis induction (PI negative, Annexin-V positive) in young C57BL/6 mice subjected to different treatments (four mice: 72 h after 0.02 Gy, eight mice: 72 h after 3 Gy, five mice: 72 h after 0.02 + 3 Gy, six mice: 4 h after 3 Gy, and four mice: 4 h after 0.02 + 3 Gy). The data show the mean (s.d.). The *p* values were determined by Welch’s *t* test or Mann–Whitney *U* test. **c** The percentages of apoptosis induction (PI negative, Annexin-V positive) in middle-aged C57BL/6 mice subjected to different treatments (five mice: 72 h after 0.02 Gy, four mice: 72 h after 3 Gy, four mice: 72 h after 0.02 + 3 Gy, six mice: 4 h after 3 Gy, and five mice: 4 h after 0.02 + 3 Gy). The data show the mean (s.d.). The *p* values were determined by Welch’s *t* test or Mann–Whitney *U* test.

Next, we examined the apoptosis percentage in the spleen, which contains radiation-sensitive lymphocytes, to understand the adaptive response at the cellular level in the spleen. Since apoptosis reaches maximum levels approximately 4 h postirradiation in splenocytes^5^, specimens were collected 4 h and 72 h after the final irradiation to compare the maximum levels of apoptosis and subsequent consequences (Figs. 1 and 4a). At 4 h post-irradiation, the apoptosis level was significantly greater in young and middle-aged C57BL/6 mice subjected to 3 Gy treatment than in those subjected to 0.02 Gy treatment. The adaptive response regarding apoptosis induction at 4 h after 0.02 Gy followed by 3 Gy was reduced in middle-aged C57BL/6 mice compared with young C57BL/6 mice (*p* = 0.019 in Fig. 4b and *p* = 0.41 in Fig. 4c). At 72 h after 3 Gy, damaged splenic lymphocytes were continuously eliminated by apoptosis in young C57BL/6 mice (*p* = 0.019 in Fig. 4b) but not in middle-aged C57BL/6 mice (*p* = 0.99 in Fig. 4c), although considerable individual differences were observed. These results suggest that lymphocytes damaged by 3 Gy tend to be retained in the spleen in middle-aged C57BL/6 mice compared with young C57BL/6 mice.

### Adaptive responses in young and middle-aged C57BL/6 mice at the intracellular molecular level

**Fig. 5:**
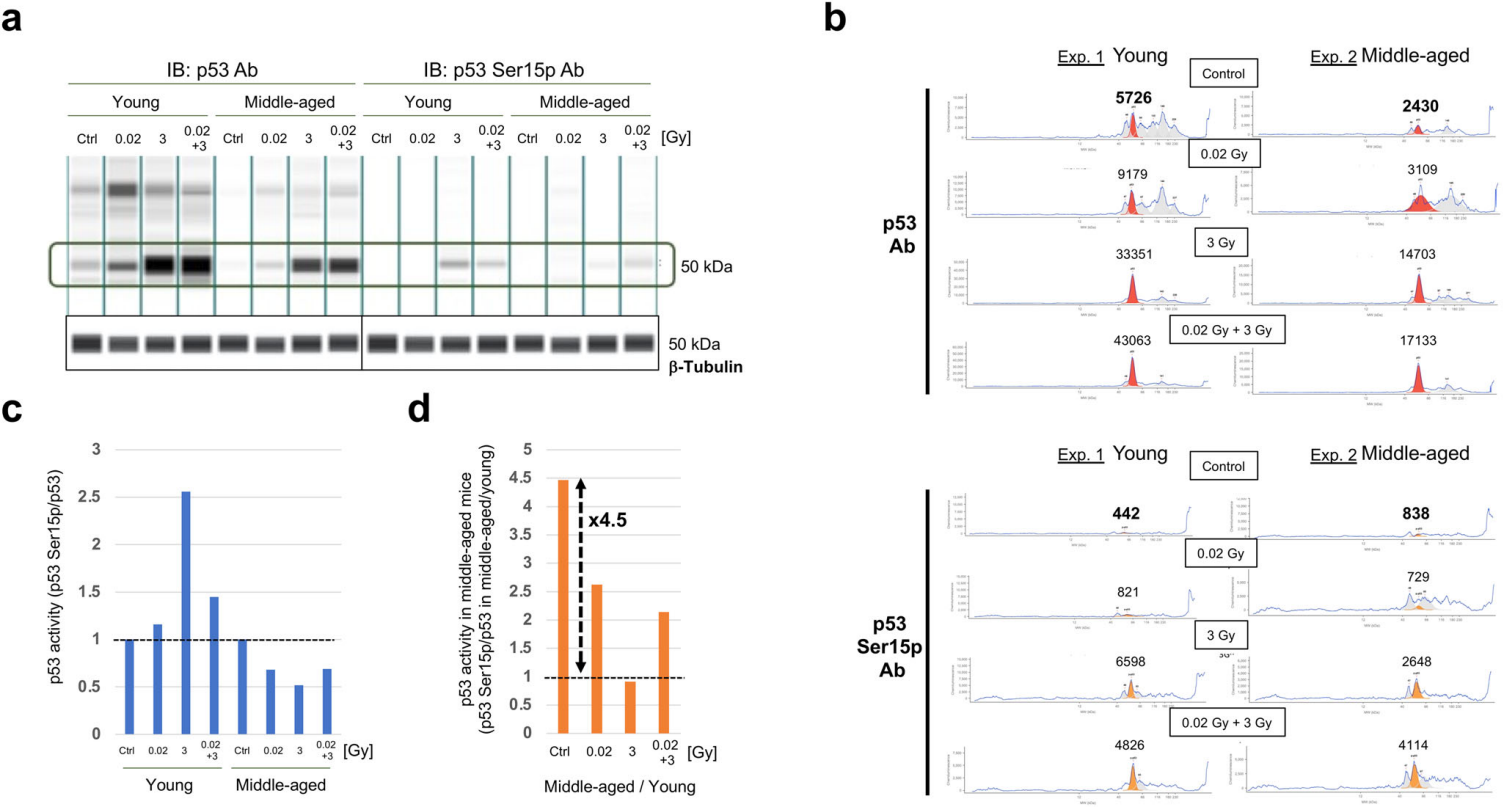
Quantification of p53 activity in the spleen at 4 h after IR by simple western blotting in experiment 1 and experiment 2. To avoid individual differences, the same amount of protein was obtained from four independent mice under each condition. **a** Simple western blotting images of p53 (left) and p53 with serine 15 phosphorylation (Ser15p, right) under four different conditions in young and middle-aged C57BL/6 mice. Simple western blotting images of β-Tubulin as determined using the same samples from four different conditions in young and middle-aged C57BL/6 mice as a loading control. **b** Quantification of p53 (upper, red) and p53 Ser15p (lower, orange) under four different conditions (control, 0.02 Gy, 3 Gy, and 0.02 + 3 Gy) in young (left) and middle-aged (right) C57BL/6 mice. **c** Relative p53 activity was calculated as the p53 Ser15p level divided by the total p53 expression level in young (left) and middle-aged (right) C57BL/6 mice. Specifically, we calculated 442/5726 (=0.077), 821/9179 (=0.089), 6598/33351 (=0.198), and 4826/43063 (=0.112) for the control group, 0.02 Gy group, 3 Gy group, 0.02 + 3 Gy group, respectively, in young mice. Then, we normalized to obtain 1 for the control group, 1.16 (0.089/0.077) for the 0.02 Gy group, 2.57 (0.198/0.077) for the 3 Gy group, and 1.45 (0.112/0.077) for the 0.02 + 3 Gy group, to assess the effects of the adaptive response. **d** As we noticed spontaneous p53 activation in middle-aged C57BL/6 mice, we calculated the relative p53 activation in middle-aged C57BL/6 mice compared with young C57BL/6 mice. Specifically, we calculated 442/5726 (=0.077) and 838/2430 (=0.345) for young and middle-aged control samples, respectively. Then, we calculated 4.48 (0.345/0.077) as the relative activation in middle-aged mice compared with young mice.

**Fig. 6:**
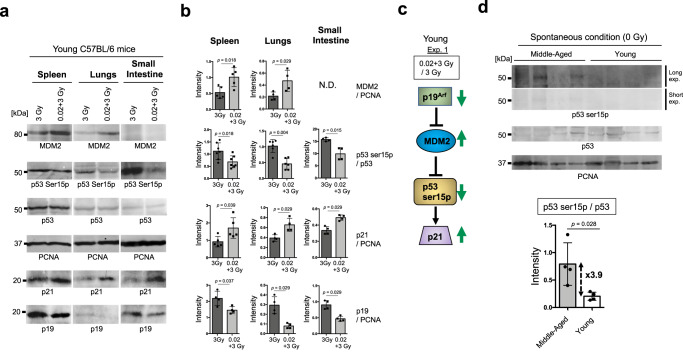
p53 activity and proteins involved upstream and downstream of the p53 pathway in the spleen, lungs and SI at 4 h post-IR exposure were quantified by standard western blotting in experiment 1. **a** Western blotting images of MDM2 (top), p53 Ser15p (second from top), p53 (third from top), PCNA (third from bottom), p21 (second from bottom), and p19^ARF^ (bottom) in young C57BL/6 mice under 3 Gy or 0.02 + 3 Gy conditions. **b** Quantification of p53 Ser15p divided by p53 under 3 Gy or 0.02 + 3 Gy conditions. Quantification of p19^ARF^, MDM2, and p21 divided by PCNA under 3 Gy or 0.02 + 3 Gy conditions. The data show the mean (s.d.). The *p* values were determined by Welch’s t test or Mann–Whitney U test. **c** Simplified model of the p53 activation pathway. In the p53 upstream pathway, MDM2 can ubiquitinate p53 for degradation by the proteome^16^, which is inhibited by p19^ARF^^15^. p21 is activated by p53^18^. Arrow: activation; Bar: inhibition. The green arrows indicate the changes in the 0.02 + 3 Gy group compared with the 3 Gy group in the young C57BL/6 mice. **d** Western blotting images of p53 Ser15p (top: long exposure; middle top: short exposure), p53 (middle bottom), and PCNA (bottom) in four independent samples from aged and young C57BL/6 mice under spontaneous conditions. All blots derive from the same experiment and were processed in parallel. Quantification of p53 Ser15p divided by p53 under spontaneous conditions. The data show the mean (s.d.). The *p* values were determined by Mann–Whitney *U* test.

To understand the adaptive responses of splenic lymphocytes at the intracellular molecular level, we quantitively evaluated p53 activation using a simple western system that enabled quantification of p53 activation at very low levels (Fig. 5a, b). In line with the findings of a reported study^5^, both p53 levels and p53 phosphorylation levels were increased by 3 Gy, while only the p53 phosphorylation level was decreased by 0.02 + 3 Gy in young C57BL/6 mice (Fig. 5a) as an adaptive response to LDR. In contrast, both p53 levels and p53 phosphorylation levels were decreased in middle-aged C57BL/6 mice compared with young C57BL/6 mice (Fig. 5a). However, the p53 phosphorylation level was slightly increased by 0.02 + 3 Gy compared with 3 Gy irradiation in middle-aged C57BL/6 mice (Fig. 5c). To confirm these adaptive response results in young C57BL/6 mice, we performed standard western blotting using proteins from cells in the spleen, lungs, and SI. We indeed observed similar results, as the p53 phosphorylation level in young C57BL/6 mice was lower in the 0.02 + 3 Gy group than in the 3 Gy group (Fig. 6a, b). In addition, the levels of upstream factors of the p53 pathway (p19^ARF^ and MDM2) correlated with p53 activation in young C57BL/6 mice (Fig. 6a–c, Supplementary Fig. 1), although the p19 expression levels were much lower in the lungs than in the spleen, and the MDM2 expression levels in the SI were below the limit of quantification. Importantly, p21, a downstream factor of p53 activation, was significantly activated in the 0.02 + 3 Gy group compared with the 3 Gy group of young C57BL/6 mice (Fig. 6a–c). Taken together, these results suggest that young mice pretreated with LDR can efficiently respond to HDR even with low p53 activation.

The p53 level was 2.36-fold lower in middle-aged C57BL/6 mice than in young C57BL/6 mice, even in spontaneous conditions (2430 in middle-aged vs. 5726 in young mice, Fig. 5b). Interestingly, we noticed that the p53 phosphorylation level was 1.89-fold higher in middle-aged C57BL/6 mice than in young C57BL/6 mice under spontaneous conditions (442 in young vs. 838 in old mice, Fig. 5b). Thus, p53 activation (phosphorylated p53/total p53) in middle-aged C57BL/6 mice was nearly 4.5-fold higher than that in young C57BL/6 mice under spontaneous conditions (Fig. 5d). To confirm that p53 is spontaneously activated in middle-aged C57BL/6 mice as found by the ProteinSimple WES system, we optimized the conditions for detection of p53 spontaneous activation for standard western blotting by using four young and four middle-aged C57BL/6 mice (Fig. 6d, Supplementary Fig. 2). Indeed, middle-aged mice had significantly higher p53 activation than young C57BL/6 mice in spontaneous condition (*p* = 0.028). Notably, the rate of spontaneous p53 activation in middle-aged C57BL/6 mice compared to young C57BL/6 mice was nearly identical between the Simple WES system and standard western blotting (4.5 in Fig. 5d vs. 3.9 in Fig. 6d). These results suggest that p53 is spontaneously activated by endogenous and exogenous stresses associated with aging. Such spontaneous p53 activation in middle-aged C57BL/6 mice may mask a series of very weak stresses that are required for hormesis effects, including lifespan extension during exposure to severe stressors^27^.

### Confirmation of an essential role of p53 in the adaptive response of young C57BL/6 mice

**Fig. 7:**
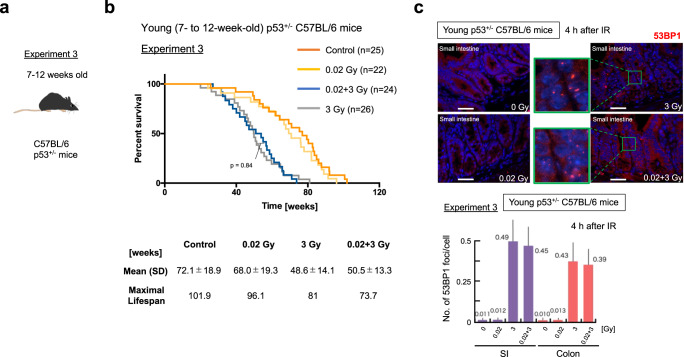
Evaluation of p53 function for adaptive responses in young C57BL/6 mice using heterogeneously p53-inactivated C57BL/6 mice. The experiments were conducted in (**a**) 7 to 12-week-old young p53^+/−^ C57BL/6 mice as experiment 3. **b** Kaplan‒Meier survival curves of experiments 3. Gehan‒Breslow‒Wilcoxon tests were used to calculate the significance of the survival curves. Each experiment was conducted several times with different numbers of mice, and the total result is shown. Time 0 indicates the point at which the mouse was born. **c** Representative images of 53BP1 foci (red) and DAPI (blue) at 4 h after IR exposure in the crypts of young p53^+/−^ C57BL/6 mice. Scale bars 0.1 mm. The enlarged images are shown in green next to the original images. The numbers of 53BP1 foci per cell at 4 h after IR exposure in young p53^+/−^ C57BL/6 mice are shown below the images. Five mice per condition were used, and data were obtained at 10 randomly selected sites. The data show the mean (s.d.). The *p* values were determined by the two-tailed *t* test.

Previously we found that the effective time interval between the priming LDR and HDR for the adaptive response can last 1 week or more in young wild-type C57BL/6 mice but is only 4 h in young p53^+/−^ C57BL/6 mice^5,6^. Therefore, we performed the 3rd experiment using young p53^+/−^ C57BL/6 mice to confirm the essential role of p53 in adaptive response for the life-elongation effects of LDR in young C57BL/6 mice (Fig. 7a). Importantly, such life extension induced by LDR was not observed in young p53^+/−^ C57BL/6 mice (*p* = 0.84, Fig. 7b). Moreover, tissue damage reductions induced by pre-LDR were not observed in young p53^+/−^ C57BL/6 mice (Fig. 7c). These results suggest that intact p53 function is indeed indispensable for adaptive responses in young C57BL/6 mice.

### Specific SASP factors are significantly associated with adaptive responses in young mice

**Fig. 8:**
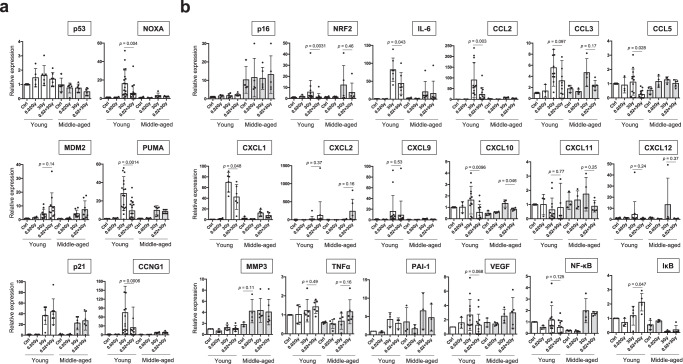
Relative mRNA expression levels of p53-related genes and SASP genes in the spleen. All mRNA expression was calculated relative to the expression of housekeeping gene (*GAPDH*), and the mRNA expression levels in young control mice are shown as 1. Each dot indicates the result for an individual mouse. The data show the mean (s.d.). The *p* values were determined by Welch’s t test or Mann–Whitney U test. **a** mRNA expression levels of p53-related factors, with *MDM2* and *p21* as direct regulators, *CCNG1* as a cell cycle factor, and *NOXA* and *PUMA* as apoptosis-inducing factors. **b** mRNA expression levels of the senescence biomarker *p16*, the hormesis-related transcription factor *NRF2*, and SASP markers (*IL-6, CCL2, CCL3, CCL5, CXCL1, CXCL2, CXCL9, CXCL10, CXCL11, MMP3, NF-κB, PAI-1, TNFα, VEGF*) along with the *NF-κB* inhibitor *IκB*.

Finally, we sought to identify the factors associated with the adaptive response for lifespan extension by examining the mRNA expression levels of SASP factors^23–26^ and factors in p53-regulatory pathways^6,19^. Consistent with the findings of a previous study^11^, the mRNA expression levels of *p53* and p53-regulated factors tended to decline with age (Fig. 8a). There were correlations between the mRNA levels and protein levels of p53 pathway members in the spleen (Figs. 5, 6, and 8a), although individual differences were also observed. Consistent with a reduction in apoptosis during the adaptive response of young mice (Fig. 4b), the mRNA expression levels of *NOXA* and *PUMA*, the apoptotic downstream transcriptional targets of p53^19^, and *CCNG1*, a cell cycle downstream transcriptional target of p53^6^, were significantly lower in the 0.02 + 3 Gy group than in the 3 Gy group (Fig. 8a).

We first confirmed that the crucial senescence biomarker p16 was expressed at a higher level in middle-aged mice than in young mice (Fig. 8b)^28^. Next, we examined a key hormesis transcription factor, *NRF2*, and the hormesis effects induced by LDR were observed only in young mice (*p* = 0.0031, Fig. 8b)^3^. Then, we investigated several key SASP factors, such as *IL-6*, *CXCL1*, and *MMP3*, among many SASP factors (Fig. 8b). Interestingly, some SASP factors (*IL-6, CCL2*, *CCL5*, *CXCL1, CXCL10*) were significantly suppressed and some SASP factors (*CCL3*, *NF-κB*, *VEGF*) were somewhat significantly suppressed in the adaptive responses in young mice, whereas other SASP factors (*CXCL2, CXCL9, CXCL11, CXCL12, MMP3*, *PAI-1, TNFα*) showed no such tendency (Fig. 8b). These results suggest that adaptive responses actively suppress specific SASPs in young C57BL/6 mice even 4 h after IR exposure.

## Discussion

It has been difficult to verify the effects of LDR^1^, but examining a phenomenon named the radiation adaptive response, which was first reported by ref. ^29^, can provide sufficient scientific data on LDR. The adaptive response is not peculiar to radiation but rather is also observed after exposure to chemical substances^7,30,31^ or nonionizing radiofrequency fields^32^; therefore, it is also known as the hormesis effect as a broad concept^3^. Assessment of adaptive responses in mice may thus be useful in assessing the health effects of exposure to environmental toxic stresses on middle-aged occupational workers, although the mechanisms of adaptive responses may differ to some extent between mice and humans. In this study, we aimed to understand the effect of LDR. In particular, we focused on middle-aged C57BL/6 mice, because most occupational workers, including radiation therapy personnel, are middle-aged.

**Fig. 9:**
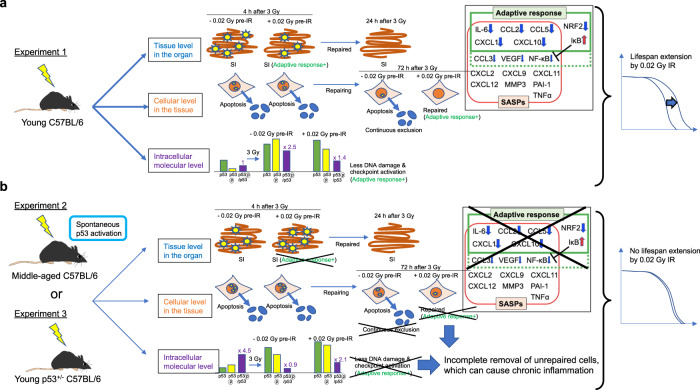
Graphical summary of the findings obtained in this study. **a** The reduced DNA damage at the tissue level (blue) and the subsequently reduced requirement of the DNA repair machinery at the intracellular molecular level (purple) may reflect adaptation to worse DNA damage in young C57BL/6 mice. In addition, continuous elimination of damaged cells by apoptosis at the cellular level in the tissue (orange) occurs in young C57BL/6 mice. Moreover, during the adaptive response in young mice, the mRNA levels of some SASP factors, such as *IL-6*, *CCL2*, and *CXCL1*, are decreased, while the mRNA levels of *IκB*, which is essential for *NFκB* suppression, are increased (boxed figure). Combined, these responses result in lifespan extension after exposure to very low doses of IR in young C57BL/6 mice (right). **b** Worse DNA damage at the tissue level (blue) but subsequent exhaustion of the DNA repair machinery at the intracellular molecular level (purple) may reflect much less adaptation to worse DNA damage in middle-aged C57BL/6 mice than in young C57BL/6 mice. Continuous elimination of damaged cells by apoptosis at the cellular level (orange) is not observed in middle-aged C57BL/6 mice. Combined, these results suggest that there is no lifespan extension induced by very low doses of IR in middle-aged C57BL/6 mice as a consequence of the chronic inflammation of unrepaired cells (right).

We first found that the life-elongation effects of LDR in young C57BL/6 mice disappeared in middle-aged C57BL/6 mice (0.02 + 3 Gy vs. 3 Gy in Fig. 2) at the organismal level (Fig. 9, right). Although the Kaplan‒Meier survival curves in this study were consistent with previously reported Kaplan‒Meier survival curves during the adaptive response^33–35^, a limitation of this study was the lack of detailed pathological diagnoses in all mice. The shorter mean lifespan of the controls in experiment 1 (103.6 ± 31.9) compared to that of the controls in experiment 2 (113.9 ± 24.9) may have been due to an experimental bias in selecting mice that survived to at least 40–62 weeks of age, as the mice in experiment 2 were irradiated at 40–62 weeks of age (Fig. 2). After this experimental bias was taken into account, there was a trend toward a slightly longer (*p* = 0.288, log-rank (Mantel–Cox) test) lifespan for middle-aged mice after 3 Gy irradiation than for younger mice after 3 Gy irradiation (Fig. 2a, b). This trend may indicate that middle-aged mice have spontaneously accumulated weak damage and are already somewhat ready for adaptive responses like 0.02 Gy pre-irradiated young mice.

Next, analyses at the tissue level (blue upper box in Fig. 9) revealed that there were fewer 53BP1 foci in the 0.02 + 3 Gy group than in the 3 Gy group in young mice (Fig. 3e), suggesting that DNA repair ability is enhanced by 0.02 Gy preirradiation at young ages^36^. In addition, apoptosis induction was attenuated (Fig. 4b). In middle-aged mice, on the other hand, the DNA repair capacity is reduced^37^, and age-related declines in p53 function were observed in the current study (Figs. 3e, 5a, 8a). We observed that the 3 Gy-induced tissue damage reflected by 53BP1 foci was promptly repaired within 24 h, particularly in the SI (Fig. 3c); such quick repair made distinguishing between the adaptive responses of the 3 Gy and 0.02 + 3 Gy groups difficult. The difference in repair ability between the SI and colon may reflect the growth activity of stem cells after DNA repair^21^. The IR-damaged tissues were surrounded by a microenvironment that might have promoted repair. Indeed, tissue can respond as a coherent unit to damage via immune system components, including cytokines and TGFβ^38^. Therefore, adaptive response experiments on damaged SI tissues that use 53BP1 foci as DSB markers should be conducted at early time points, such as 4 h (Fig. 3b).

Lymphocytes in the spleen are very sensitive to IR^39^, and the spleen shrinks rapidly after IR exposure. p53 can induce apoptosis after IR through direct transcriptional activation of *PUMA* and *NOXA*^19^. Indeed, rapid apoptosis induction at 4 h after 3 Gy was observed in young and middle-aged C57BL/6 mice (Fig. 4b, c) at the cellular level in the spleen. This apoptosis induction was decreased in 0.02 Gy-pretreated young C57BL/6 mice (*p* = 0.019, Fig. 4b) but was decreased to a lesser extent in middle-aged C57BL/6 mice (*p* = 0.41, Fig. 4c), confirming that splenocyte apoptosis can be used to detect the adaptive response. These results were confirmed by assessment of the mRNA expression levels of *PUMA* and *NOXA* in qPCR experiments (Fig. 8a). Although there were individual differences, continuous apoptosis induction was observed in the spleens of young C57BL/6 mice even 72 h after 3 Gy treatment. As a result, much less apoptosis induction was detected in young C57BL/6 mice treated with 0.02 + 3 Gy, while almost no change was detected in middle-aged C57BL/6 mice. Thus, middle-aged C57BL/6 mice failed to induce apoptosis to eliminate damaged cells 72 h after 3 Gy, resulting in chronic inflammation and an increased chance of carcinogenesis, as previously reported^40^. However, recent studies have reported that mice lacking mediators critical for p53-induced apoptosis do not spontaneously develop cancers^19^. We still do not know the precise role of p53 in apoptosis during cancer progression. For example, it remains unclear how p53 mutation can affect apoptosis induced by wild-type p53 in nonmalignant cells, nascent neoplastic cells, and malignant cells. In addition, the role of p53 in apoptosis under physiological conditions remains elusive. Our results suggest that understanding the differences in continuous apoptosis induction in the spleen between young and middle-aged C57BL/6 mice would be useful for unraveling the individual consequences of the adaptive response (orange middle box in Fig. 9).

The activity of p53 can be obtained by dividing the expression level of Ser15-phosphorylated p53 by the total p53 expression level^41^. By using a simple western system with proteins of spleen lymphocytes, we reproduced the results regarding the adaptive response obtained by standard western blotting in young C57BL/6 mice^5^. Standard western blotting showed similar results regarding p53 activation (Fig. 6). Moreover, the significant p21 activation in the 0.02 + 3 Gy group compared with the 3 Gy group in young C57BL/6 mice (Fig. 6a–c) may have reflected efficient suppression of apoptosis in the spleen in the 0.02 + 3 Gy group compared with the 3 Gy group (Fig. 4b), given that p21 can inhibit apoptosis^42^. Coordinated p53 regulation (p19, MDM2, and p21) was observed in different organs, such as the spleen, lungs, and SI (Fig. 6a–c). Thus, analyzing these organs at the molecular level can enhance our understanding of the biological adaptive response to IR that determines the fate of the mouse. Furthermore, the requirement for intact p53 function for the lifespan-extending adaptive response was confirmed by experiments on young heterogenous p53-knockout mice (Fig. 7), suggesting that p53 is one of the most important factors for the hormesis effect. Although optimizing the experimental conditions is technically challenging, it would be interesting to use pharmacological modulation of p53 activity, for example, treatment with the MDM2 inhibitor RG711^43^, to assess the biological effects of adaptive responses. However, it should be noted that overstabilization of p53 has been reported to accelerate aging and shorten lifespan^44^.

Slightly greater p53 activation was observed in middle-aged C57BL/6 mice treated with 0.02 + 3 Gy than in middle-aged C57BL/6 mice treated with 3 Gy (Fig. 5a). This result suggests that VLDR may additively affect but not adaptively affect middle-aged C57BL/6 mice subsequently subjected to HDR exposure due to p53 functional exhaustion. Notably, quantification of very low amounts of p53 and p53 phosphorylation under spontaneous conditions enabled us to uncover an inverse relationship between young and middle-aged C57BL/6 mice. As a result, spontaneous p53 activation, which was approximately 4.5-fold stronger in middle-aged C57BL/6 mice than in young C57BL/6 mice, was unexpectedly detected by simple western blotting (dotted double arrow in Fig. 5d, purple lower box in Fig. 9). Optimized standard western blotting also revealed 3.9-fold higher spontaneous p53 activation in middle-aged C57BL/6 mice than in young C57BL/6 mice (Fig. 6d). Spontaneous p53 activation in middle-aged C57BL/6 mice can be induced by exogenous and endogenous stress factors^45^. Indeed, more 53BP1 foci per cell (0.067) were counted in the SIs of middle-aged C57BL/6 mice than in those of young C57BL/6 mice (0.013) under the 0 Gy condition (Fig. 3e). Spontaneous p53 activation by endogenous and exogenous stress may induce excessive use of p53, which may accelerate p53 exhaustion. Therefore, p53 function may decline with age, and more than half of cancer patients likely lose p53 function as a consequence. Many posttranscriptional modifications of p53 and other factors occur after DNA damage induction^46,47^, and posttranscriptional modifications other than p53 Ser15 phosphorylation may impact the adaptive response induced by LDR. p53 is a well-known pleiotropic gene associated with longevity and premature aging^48,49^, and further studies will be required to understand the positive or negative roles of p53 in the adaptive response induced by VLDR.

Importantly, some SASP factors (*IL-6, CCL2, CCL5*, *CXCL1, CCL10*) were significantly suppressed and some SASP factors (*CCL3*, *NF-κB*, *VEGF*) were slightly significantly suppressed during the adaptive response in young mice^23–26^, suggesting that specific SASP factors are involved in the radiation adaptive response (Figs. 8, 9). Of note, significantly higher *IκB* expression was observed in young mice in the 0.02 + 3 Gy group than in young mice in the 3 Gy group; however, this phenotype was completely absent in the middle-aged mice, in which extended survival was not observed (Fig. 8b). These data suggest that the IκB-related signaling pathway, which is essential for suppression of the activity of the important inflammation factor NF-κB^50^ may be one of the important pathways for the adaptive response in young mice. Given that p53 actively represses the SASP^26^ and that p53 mRNA and protein levels are lower in middle-aged mice than in young mice (Figs. 5 and 8), the SASP is likely upregulated in middle-aged mice. However, *IL-6*, *CCL2*, *CXCL1*, and *CXCL10* levels were not elevated after IR exposure in middle-aged mice (Fig. 8b), suggesting that aging decreases the overall function of DNA damage sensors/transducers upstream of both the p53 pathway and the SASP pathway^25^.

Currently, the biological effects of VLDR remain largely unknown due to technical difficulties. However, the need to understand the biological effects induced by VLDR is quite urgent for the majority of middle-aged occupational workers. Therefore, we tried to systematically elucidate the biological effects of VLDR at different levels in young and middle-aged C57BL/6 mice, which are the most popular experimental mice to date. This study uncovered spontaneous p53 activation in middle-aged C57BL/6 mice, which may reflect forced p53 exhaustion and could be responsible for the diminished adaptive response for lifespan extension compared with that of young C57BL/6 mice. Thus, these results may suggest the importance of detecting spontaneous p53 activation in the middle-aged population as a useful marker for assessing healthy aging and responses to unexpected accidents in the future.

## Methods

### Experimental animals

Wild-type C57BL/6 N mice of the parental wild-type inbred strain were obtained from Japan Charles River Laboratories Inc. (Yokohama, Japan). Mice with a nonfunctional *p53* gene (*p53*^−/−^ mice) were obtained and maintained as described previously^5^. *p53*^*+/−*^ mice were obtained by crossing *p53*^*−/−*^ mice with *p53*^*+/+*^ mice. The mice were housed in rooms with a controlled temperature (22 ± 1.5 °C) and humidity (50 ± 10%) and a standard 12-h light/12-h dark cycle in a specific pathogen-free environment. The mice were maintained on sterile water and a standard pellet diet (MF, Oriental Yeast Co., Ltd). All care and use of the animal subjects followed the stipulations of the ARRIVE guidelines, Laboratory Animal Research Center and the Use Committee of the University of Occupational and Environmental Health, Japan. The experimental protocols were approved by the Ethics Review Committee for Animal Experimentation of the University of Occupational and Environmental Health, Japan (AE13-008).

### Irradiation of mice

Priming irradiation (0.02 Gy (667 mGy/min)) was applied to mice by whole-body irradiation using an SK-951 ^137^Cs g-ray irradiator (Sangyo Kagaku Inc, Tokyo, Japan). After waiting periods of 72 h, the mice were then irradiated with a 3 Gy (0.72 Gy/min) challenge dose from a ^137^Cs Gammacell 40 Exactor (MDS Nordion, Ottawa, Canada). The experimental groups included a control group (sham irradiation) and groups that received either 0.02 Gy or 3 Gy alone.

### Survival analysis and histological evaluation

Survival was monitored until all mice died after irradiation. Moribund mice were euthanized via cervical dislocation and necropsied. Kaplan‒Meier survival analysis was performed. The excised mouse tissues were fixed in 10% neutral-buffered formalin, dehydrated, and embedded in paraffin by conventional methods. Subsequently, 5-µm sections were cut from formalin-fixed paraffin-embedded blocks and transferred to glass slides. The sections were stained with hematoxylin and eosin (H&E) using standard procedures.

### Immunofluorescence staining

Immunofluorescence was performed to quantitatively analyze 53BP1 foci, which are representative DSB markers^22^, in Ki-67-positive proliferating cells in the intestines and to assess the kinetics of DNA damage repair and the elimination of damaged cells. The proximal (duodenum) and distal (ileum) SIs and the colons from C57BL/6 N mice were fixed in 4% neutral-buffered paraformaldehyde. Three-micrometer sample sections were deparaffinized, rehydrated and heat-reactivated for antigen retrieval. The following antibodies were used for the immunofluorescence staining: anti-Ki-67 (1:1000 dilution; 652402; BioLegend); anti-53BP1 (1:2000 dilution; A300-272A; Bethyl Laboratories); Alexa Fluor 546- and 647-conjugated goat anti-rabbit IgG and anti-mouse IgG (1:2000 dilution; A-11071, A-21246, A-21123, 405322, Thermo Fisher Scientific). The samples were analyzed using widefield microscopy (F3000B fluorescence microscope; Leica Microsystems), and digital images were captured and analyzed using FW4000 software (Leica Microsystems). Z-stack images were acquired every 0.5 µm at 400× magnification, and deconvoluted images were used to create two-dimensional images. The optimal intensity threshold, exposure time and gain were set to minimize background noise, and these settings were kept constant during the experiments. Fluorescence signals greater than threefold higher than the background with Ki-67 signals in the nuclei were considered Ki-67-positive. For quantification of Ki-67-positive cells and 53BP1 foci per cell, at least 100 crypts were examined. More than fifteen 53BP1 foci per cell were observed immediately (10 min) after 3 Gy exposure. Quantitative analysis was technically difficult; therefore, the number of cells was calculated according to the intensity of DAPI (DAPI FL), and the number of 53BP1 foci was calculated according to the intensity of the red color (RED FL). Specifically, the 53BP1 foci were quantified according to the ratio of RED FL to DAPI FL.

### Annexin-V assay for apoptotic cells in the mouse spleen

An Annexin V-FITC assay kit (4700, MBL, MEBCYTO Apoptosis Kit) was used to detect apoptotic cells in the mouse spleen after IR exposure according to the manufacturer’s instructions. Irradiated mice were sacrificed at 4 h or 72 h after IR exposure by cervical dislocation. Mouse spleens were placed into PBS in 35-mm dishes on ice and minced using the frosted part of a glass slide. After filtering with 100-µm mesh to eliminate aggregated cells, 1–3 × 10^5^ splenocytes were collected by centrifugation at 500 × *g* for 3 min at 4 °C. The cells were resuspended in 85 μL of binding buffer; next 10 μL of Annexin V-FITC and 5 μL of propidium iodide (PI) were added, and the cells were incubation at room temperature (20–25 °C) for 15 min in the dark. Then, 400 μL of binding buffer was added, and the cells were analyzed by CytoFLEX (Beckman Coulter). Annexin V-FITC-positive and PI-negative cells were considered apoptotic cells.

### Western blotting

Irradiated or nonirradiated mice were sacrificed at 4 h after the final IR exposure by cervical dislocation. Small pieces of spleen, lungs, and SI were excised, placed into Eppendorf tubes, frozen in liquid nitrogen, and stored in a −80 °C freezer. The frozen tissues were disrupted with a cold homogenizer pestle (2-4478-02, Asone). The minced tissues were treated with 200 µL of RIPA buffer (50 mM Tris, pH 7.5, 150 mM NaCl, 1% Triton-X, 0.5% sodium deoxycholate, 0.1% SDS) supplemented with protease inhibitor (05892791001, Roche) and phosphatase inhibitor (4906845001, Roche) on ice for 30 min with occasional vortexing. The samples were sonicated (6 cycles of 10 s on and 10 s off; TAITEC, VP-15S, Saitama) on ice and centrifuged for 20 min at 23,000 g at 4 °C.

### (a) Automated capillary-based western blotting system

The supernatant was transferred to new tubes and applied to a ProteinSimple WES system according to the manufacturer’s instructions. We used four independent mice for each condition, and the proteins collected from four mice with the same protein quantity were combined and used for simple western blotting as a representative western blotting sample to exclude individual differences. The protein levels of p53 and p53 phosphorylated at Ser15 (Ser15p) were quantified using an automated capillary-based western blotting system (WES, ProteinSimple, Japan). All steps were performed with the manufacturer’s reagents according to the user manual. A total of 4 μl of cell lysate was mixed with 1 μl of 5x fluorescent master mix with DTT included in the kit and heated at 95 °C for 5 min. The cell lysates, a biotinylated ladder, primary and secondary antibodies, and HRP chemiluminescent substrate were dispensed into designated wells in the assay plate included in the kit. All steps, including separation, stacking and immobilization, were automatically performed using a separation matrix for low-molecular-weight proteins (Standard Pack 1, 12–230 kDa; ProteinSimple). The data were analyzed using Compass software, which was already installed in the device, according to the ProteinSimple protocol. The primary antibodies used in this study were anti-p53 (1:100 dilution; ab1101, Abcam) and anti-phospho-p53 (Ser15) (1:100 dilution; 9284, Cell Signaling).

### (b) Standard western blotting

Alternatively, the supernatant was transferred to new tubes and boiled with sample buffer (100 mM Tris-HCl [pH 6.8], 100 mM DTT, 4% SDS, 20% glycerol, and 0.2% bromophenol blue) for 5 min at 95 °C, and 20–40 µg of protein was used for standard western blotting. The primary antibodies used in this study were anti-p53 (1:1000 dilution; ab1101, Abcam), anti-phospho-p53 (Ser15) (1:1000 dilution; 9284, Cell Signaling), anti-p19 INK4d (1:1000 dilution; 10272-2-AP, Proteintech), anti-p21 (1:1000 dilution; 10355-1-AP, Proteintech), anti- MDM2 (1:1000 dilution; sc-965, Santa Cruz), anti-β-Tubulin (1:2000 dilution; 014-25041, Wako), and anti-PCNA (1:2000 dilution; sc-56, Santa Cruz). The latter two antibodies served as the loading controls. Generally, we used 1% skim milk (190-12865, Wako) in TBS-T for blocking; however, 1% BSA (10735086001, Roche) in TBS-T was required for the detection of p53 phosphorylation in spontaneous conditions. The membranes were washed three times with TBS-T at room temperature for 10 min and treated with goat-anti-mouse IgG or goat-anti-rabbit IgG-AP (1:2000 dilution; A3812, A3688, Sigma-Aldrich) secondary antibodies. BCIP/NBT (S3771, Promega) was used to develop the blots. The band intensities were analyzed with ImageJ-3 software. All blots were derived from the same experiment and were processed in parallel. Blots from liver and kidney tissues were not used because they did not give clean results.

### Quantification of mRNA expression levels by real-time qPCR

RNA was extracted from mouse spleens using QIAzol Lysis Reagent (79306, Qiagen, Hilden, Germany) followed by chloroform treatment. The RNA was converted into cDNA using a ReverTra Ace qPCR RT Kit (FSQ-101, TOYOBO, Osaka, Japan) or SuperScript IV VILO Master Mix with ezDNase Enzyme (11766050, ThermoFisher Scientific). Expression levels were analyzed with a StepOnePlus qPCR system or a QuantStudio 3/5 Real-Time PCR system (Thermo Fisher Scientific) with THUNDERBIRD SYBR qPCR Mix (QPS-201, TOYOBO, Osaka, Japan). A complete list of primers used for Real-Time qPCR, including names and sequences, is provided in Supplementary Information.

### Statistics

All statistical tests were two-sided. *P* values of 0.05 or less were used to denote statistical significance. No statistical methods were used to predetermine the sample size. For two-group comparisons, the data were analyzed by a Kolmogorov–Smirnov test to assess the normality of the distribution. We rejected the null hypothesis if *p* < 0.05. Normally distributed data were analyzed using Welch’s *t* test. Data with a nonnormal distribution were analyzed using the Mann–Whitney *U* test. The p values for the 53BP1 foci in intestines were determined using two-tailed *t* test; the *p* values for the Kaplan‒Meier survival analysis were determined using the Log-rank (Mantel–Cox) test or the Gehan‒Breslow‒Wilcoxon test. All analyses were performed using GraphPad Prism (version 6.0 g).

### Reporting Summary

Further information on research design is available in the Nature Research Reporting Summary linked to this article.

### Supplementary information

Supplementary information

reporting-summary

## Data Availability

All data related to this study are available in the article.
