# Supplementary material for: Spontaneous p53 activation in middle-aged C57BL/6 mice mitigates the lifespan-extending adaptive response induced by low-dose ionizing radiation
Source: NPJ Aging. 2023 Nov 7;9(1):26. doi: 10.1038/s41514-023-00123-3 (PMC10630390; doi:10.1038/s41514-023-00123-3)
Supplement: Supplementary file 1 — Supplementary information [file 41514_2023_123_MOESM1_ESM.pdf]

# Supplementary information

## Spontaneous p53 activation in middle-aged C57BL/6 mice mitigates the lifespan-extending adaptive response induced by low-dose ionizing radiation

**Masaoki Kohzaki, Keiji Suzuki, Akira Ootsuyama, Ryuji Okazaki**

University of Occupational and Environmental Health, Kitakyushu, Japan
Nagasaki University, Nagasaki, Japan

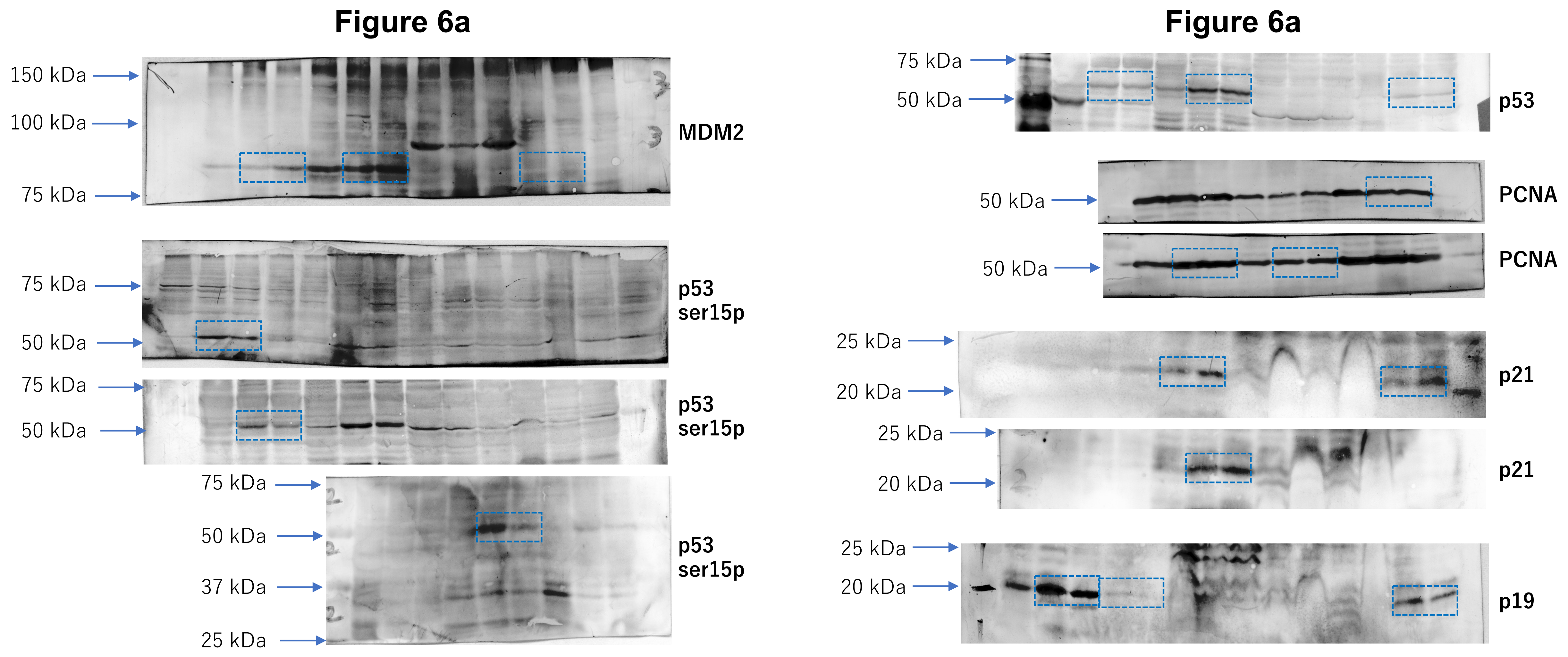


Supplementary Figure 1. Unprocessed Western blots. The original scans of the most important Western blots for Figures 6a are shown. Blue dotted rectangles of the original scans mark the position of lanes that are relevant to the figure in the main text. Arrowhead mark the position of protein markers. We did not use liver or kidney data because samples from liver or kidney tissues did not give clean results.

# Figure 6d

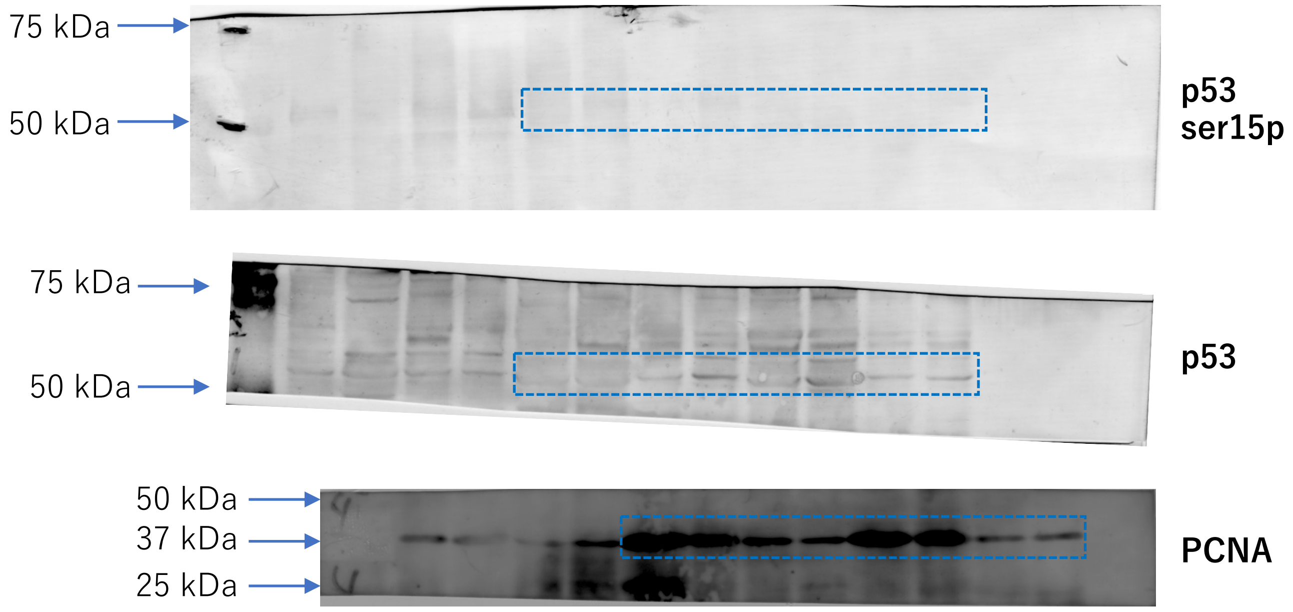


Supplementary Figure 2. Unprocessed Western blots. The original scans of the most important Western blots for Figures 6d are shown. Blue dotted rectangles of the original scans mark the position of lanes that are relevant to the figure in the main text. Arrowhead mark the position of protein markers.

# Supplementary information

## Primer pairs for RT-qPCR

| Gene name | Sequence of forward primer | Sequence of reverse primer |
| --- | --- | --- |
| **GAPDH** | GGTGCTGAGTATGTCGTGGA | CGGAGATGATGACCCTTTTG |
| **p53** | ATGGCCATCTACAAGAAGTCACAG | ATCGGAGCAGCGCTCATG |
| **MDM2** | GGACTCGGAAGATTACAGCCTGA | TGTCTGATAGACTGTGACCCG |
| **p21** | AGATCCACAGCGATATCCAGAC | ACCGAAGAGACAACGGCACACT |
| **NOXA** | TCGCAAAAGAGCAGGATGAG | CACTTTGTCTCCAATCCTCCG |
| **PUMA** | ACGACCTCAACGCGCAGTACG | GAGGAGTCCCATG AAGAGATTG |
| **CCNG1** | CGTGTCCTCAGTTCTTTGGCTTTGACACG | GATGCTTCGCCTGTACCTTCATT |
| **p16** | AGGGCCGTGTGCATGACGTG | GCACCGGGCGGGAGAAGGTA |
| **IL-6** | CCTCTCTGCAAGAGACTTCCAT | AGTCTCCTCTCCGGACTTGT |
| **CXCL1** | GCTGGGATTCACCTCAAGAA | AGGTGCCATCAGAGCAGTCT |
| **MMP3** | GTCCCTCTATGGAACTCCCAC | AGTCCTGAGAGATTTGCGCC |
| **NF-κB** | GAAATTCCTGATCCAGACAAAAAC | ATCACTTCAATGGCCTCTGTGTAG |
| **IκB** | TCAGCATGAGCCCTTCCTGGAT | CAAGGATGGCTGCTAGATGCAG |
| **TNFα** | AGCACAGAAAGCATGATCCG | GTTTGCTACGACGTGGGCTA |
| **NRF2** | CAGCATAGAGCAGGACATGGAG | GAACAGCGGTAGTATCAGCCAG |
| **VEGF** | CTGTGCAGGCTGCTGTAACG | GTTCCCGAAACCCTGAGGAG |
| **PAI-1** | ACGCCTGGTGCTGGTGAATGC | ACGGTGCTGCCATCAGACTTGTG |
| **CCL2** | CCCAATGAGTAGGCTGGAGA | TCTGGACCCATTCCTTCTTG |
| **CCL3** | ATGAAGGTCTCCACCACTGCCCTTGC | TCAGGCATTCAGTTCCAGGTCAGTGA |
| **CCL5** | ATGAAGATCTCTGCAGCTGCCCTC | CTAGCTCATCTCCAAATAGTTGATG |
| **CXCL2** | CGCTGTCAATGCCTGAAG | GGCGTCACACTCAAGCTCT |
| **CXCL9** | GGAACCCTAGTGATAAGGAATGCA | TGAGGTCTTTGAGGGATTTGTAGTG |
| **CXCL10** | GCCGTCATTTTCTGCCTCA | CGTCCTTGCGAGAGGGATC |
| **CXCL11** | AACAGGAAGGTCACAGCCATAGC | TTTGTCGCAGCCGTTACTCG |
| **CXCL12** | GTCCTCTTGCTGTCCAGCTC | TTTCAGATGCTTGACGTTGG |
