# Supplementary material for: Spontaneous p53 activation in middle-aged C57BL/6 mice mitigates the lifespan-extending adaptive response induced by low-dose ionizing radiation
Source: NPJ Aging. 2023 Nov 7;9(1):26. doi: 10.1038/s41514-023-00123-3 (PMC10630390; doi:10.1038/s41514-023-00123-3)
Supplement: Supplementary file 2 — reporting-summary [file 41514_2023_123_MOESM2_ESM.pdf]

nature portfolio

Corresponding author(s): Masaoki Kohzaki

Last updated by author(s): Sep 15, 2023

Reporting Summary

Nature Portfolio wishes to improve the reproducibility of the work that we publish. This form provides structure for consistency and transparency in reporting. For further information on Nature Portfolio policies, see our Editorial Policies and the Editorial Policy Checklist.

Statistics

For all statistical analyses, confirm that the following items are present in the figure legend, table legend, main text, or Methods section.

n/a	Confirmed	
☐	☒	The exact sample size (*n*) for each experimental group/condition, given as a discrete number and unit of measurement
☐	☒	A statement on whether measurements were taken from distinct samples or whether the same sample was measured repeatedly
☐	☒	The statistical test(s) used AND whether they are one- or two-sided<br>*Only common tests should be described solely by name; describe more complex techniques in the Methods section.*
☒	☐	A description of all covariates tested
☐	☒	A description of any assumptions or corrections, such as tests of normality and adjustment for multiple comparisons
☐	☒	A full description of the statistical parameters including central tendency (e.g. means) or other basic estimates (e.g. regression coefficient) AND variation (e.g. standard deviation) or associated estimates of uncertainty (e.g. confidence intervals)
☐	☒	For null hypothesis testing, the test statistic (e.g. *F*, *t*, *r*) with confidence intervals, effect sizes, degrees of freedom and *P* value noted<br>*Give P values as exact values whenever suitable.*
☒	☐	For Bayesian analysis, information on the choice of priors and Markov chain Monte Carlo settings
☒	☐	For hierarchical and complex designs, identification of the appropriate level for tests and full reporting of outcomes
☒	☐	Estimates of effect sizes (e.g. Cohen's *d*, Pearson's *r*), indicating how they were calculated

Our web collection on statistics for biologists contains articles on many of the points above.

Software and code

Policy information about availability of computer code

Data collection	All data collection were performed using Microsoft Excel (version 16.54).
Data analysis	All analyses were performed using GraphPad Prism (version 6.0 g).

For manuscripts utilizing custom algorithms or software that are central to the research but not yet described in published literature, software must be made available to editors and reviewers. We strongly encourage code deposition in a community repository (e.g. GitHub). See the Nature Portfolio guidelines for submitting code & software for further information.

Data

Policy information about availability of data

All manuscripts must include a data availability statement. This statement should provide the following information, where applicable:

- Accession codes, unique identifiers, or web links for publicly available datasets
- A description of any restrictions on data availability
- For clinical datasets or third party data, please ensure that the statement adheres to our policy

All data related to this study are available in the article.

Research involving human participants, their data, or biological material

Policy information about studies with human participants or human data. See also policy information about sex, gender (identity/presentation), and sexual orientation and race, ethnicity and racism.

Reporting on sex and gender	not applicable
Reporting on race, ethnicity, or other socially relevant groupings	not applicable
Population characteristics	not applicable
Recruitment	not applicable
Ethics oversight	not applicable

Note that full information on the approval of the study protocol must also be provided in the manuscript.

Field-specific reporting

Please select the one below that is the best fit for your research. If you are not sure, read the appropriate sections before making your selection.

☒ Life sciences ☐ Behavioural & social sciences ☐ Ecological, evolutionary & environmental sciences

For a reference copy of the document with all sections, see nature.com/documents/nr-reporting-summary-flat.pdf

Life sciences study design

All studies must disclose on these points even when the disclosure is negative.

Sample size	22-44 mice per group.
Data exclusions	We did not exclude data.
Replication	Experiments have been conducted in multiple independent cohorts with similar results.
Randomization	The experiment was performed on randomly selected mice.
Blinding	Blindness was ensured by assigning numbers to the mice to be analyzed, conducting the experiment, and matching the results after they were available.

Reporting for specific materials, systems and methods

We require information from authors about some types of materials, experimental systems and methods used in many studies. Here, indicate whether each material, system or method listed is relevant to your study. If you are not sure if a list item applies to your research, read the appropriate section before selecting a response.

Materials & experimental systems

n/a	Involved in the study	
☐	☒	Antibodies
☒	☐	Eukaryotic cell lines
☒	☐	Palaeontology and archaeology
☐	☒	Animals and other organisms
☒	☐	Clinical data
☒	☐	Dual use research of concern
☒	☐	Plants

Methods

n/a	Involved in the study	
☒	☐	ChIP-seq
☐	☒	Flow cytometry
☒	☐	MRI-based neuroimaging

Antibodies

Antibodies used	anti-Ki-67 (652402; BioLegend); anti-53BP1 (A300-272A; Bethyl Laboratories); Alexa Fluor 546- and 647-conjugated goat anti-rabbit IgG and anti-mouse IgG (A-11071, A-21246, A-21123, 405322, Thermo Fisher Scientific),anti-p53 (ab1101, Abcam), anti-phospho-p53 (Ser15) (9284, Cell Signaling), anti-p19 INK4d (10272-2 AP, Proteintech), anti-p21 (10355-1-AP, Proteintech), anti- MDM2 (sc-965,

	Santa Cruz), anti-β-Tubulin (014-25041, Wako), anti-PCNA (sc-56, Santa Cruz).
Validation	All antibodies are validated by having been used and published by many researchers.

Animals and other research organisms

Policy information about studies involving animals; ARRIVE guidelines recommended for reporting animal research, and Sex and Gender in Research

Laboratory animals	Young (7-to 12-week-old) and middle-aged (40-to 62-week41 old) C57BL/6 mice.
Wild animals	The study did not involve wild animals.
Reporting on sex	Both sex were evenly used.
Field-collected samples	The study did not involve samples collected from the field.
Ethics oversight	Laboratory Animal Research Center and the Use Committee of the University of Occupational and Environmental Health, Japan.

Note that full information on the approval of the study protocol must also be provided in the manuscript.

Flow Cytometry

Plots

Confirm that:

☒ The axis labels state the marker and fluorochrome used (e.g. CD4-FITC).

☒ The axis scales are clearly visible. Include numbers along axes only for bottom left plot of group (a 'group' is an analysis of identical markers).

☒ All plots are contour plots with outliers or pseudocolor plots.

☒ A numerical value for number of cells or percentage (with statistics) is provided.

Methodology

Sample preparation	Mouse spleens were placed into PBS in 35-mm dishes on ice and minced using the frosted part of a glass slide. After filtering with 100-μm mesh to eliminate aggregated cells, 1-3 × 105 splenocytes were collected by centrifugation at 500 × g for 3 min at 4°C. The cells were resuspended in 85 μL of binding buffer; next 10 μL of Annexin V-FITC and 5 μL of propidium iodide (PI) were added, and the cells were incubation at room temperature (20-25°C) for 15 min in the dark. Then, 400 μL of binding buffer was added.
Instrument	The cells were analyzed by 467 CytoFLEX (Beckman Coulter).
Software	Flow Jo was used to analyze data.
Cell population abundance	Cells were not sorted and similarly prepared for Annexin V analysis.
Gating strategy	No specific gating other than recognizing splenocytes with FSC and SSC.

☒ Tick this box to confirm that a figure exemplifying the gating strategy is provided in the Supplementary Information.
